# *RcTGA1 and glucosinolate biosynthesis pathway* involvement in the defence of rose against the necrotrophic fungus *Botrytis cinerea*

**DOI:** 10.1186/s12870-021-02973-z

**Published:** 2021-05-17

**Authors:** Penghua Gao, Hao Zhang, Huijun Yan, Qigang Wang, Bo Yan, Hongying Jian, Kaixue Tang, Xianqin Qiu

**Affiliations:** 1 Flower Research Institute, Yunnan Academy of Agricultural Sciences/National Engineering Research Center for Ornamental Horticulture, Kunming, 650205 China; 2 Southwest Forestry University, Kunming, 650024 China

**Keywords:** Fungus–plant interactions, Resistance genes, MAPK signalling pathway-plant, Plant hormone signal transduction, Transcriptome, VIGS, Metabolome

## Abstract

**Background:**

Rose is an important economic crop in horticulture. However, its field growth and postharvest quality are negatively affected by grey mould disease caused by *Botrytis c.* However, it is unclear how rose plants defend themselves against this fungal pathogen. Here, we used transcriptomic, metabolomic and VIGS analyses to explore the mechanism of resistance to *Botrytis c.*

**Result:**

In this study, a protein activity analysis revealed a significant increase in defence enzyme activities in infected plants. RNA-Seq of plants infected for 0 h, 36 h, 60 h and 72 h produced a total of 54 GB of clean reads. Among these reads, 3990, 5995 and 8683 differentially expressed genes (DEGs) were found in CK vs. T36, CK vs. T60 and CK vs. T72, respectively. Gene annotation and cluster analysis of the DEGs revealed a variety of defence responses to *Botrytis c.* infection, including resistance (R) proteins, MAPK cascade reactions, plant hormone signal transduction pathways, plant-pathogen interaction pathways, Ca^2+^ and disease resistance-related genes. qPCR verification showed the reliability of the transcriptome data. The PTRV2-RcTGA1-infected plant material showed improved susceptibility of rose to *Botrytis c.* A total of 635 metabolites were detected in all samples, which could be divided into 29 groups. Metabonomic data showed that a total of 59, 78 and 74 DEMs were obtained for T36, T60 and T72 (T36: *Botrytis c.* inoculated rose flowers at 36 h; T60: *Botrytis c.* inoculated rose flowers at 60 h; T72: *Botrytis c.* inoculated rose flowers at 72 h) compared to CK, respectively. A variety of secondary metabolites are related to biological disease resistance, including tannins, amino acids and derivatives, and alkaloids, among others; they were significantly increased and enriched in phenylpropanoid biosynthesis, glucosinolates and other disease resistance pathways. This study provides a theoretical basis for breeding new cultivars that are resistant to *Botrytis c.*

**Conclusion:**

Fifty-four GB of clean reads were generated through RNA-Seq. R proteins, ROS signalling, Ca^2+^ signalling, MAPK signalling, and SA signalling were activated in the Old Blush response to *Botrytis c. RcTGA1* positively regulates rose resistance to *Botrytis c*. A total of 635 metabolites were detected in all samples. DEMs were enriched in phenylpropanoid biosynthesis, glucosinolates and other disease resistance pathways.

**Supplementary Information:**

The online version contains supplementary material available at 10.1186/s12870-021-02973-z.

## Background

Rose is one of the four cut flowers in the world with high ornamental and economic value. Black spot, aphid and grey mould are three major diseases that affect rose production along with insect pests. Grey mould mainly occurs in the late flowering or postharvest period of roses. This will cause an approximate 15% ~ 40% reduction in production, and postharvest corruption will lead to serious economic losses to the cut rose market.

Grey mould of rose is caused by *Botrytis cinerea*, which belongs to *Sclerotinia* [1, 2]. *Botrytis c.* is a typical necrotrophic pathogenic fungus that infects host cells by regulating the programmed death pathway [3]. It usually infects the plant tissue at an early stage and stays for a long time. When the environment is suitable or the host physiological changes, it will burst out suddenly, leading to deterioration and decay of plant tissue. Chemical methods are mainly used to control *Botrytis c.,* such as imidazole, dicarboximides, and anilinopyrimidines. However, due to the long-term use of chemical drugs, *Botrytis c.* has developed tolerance to them. The worse the effect, the larger the drug dosage. It not only increases the economic cost but also aggravates environmental pollution. Therefore, it is urgent to find an effective method to enhance the resistance of Chinese rose to fungi.

With the development of biotechnology, whole-genome sequencing of many plant species has been completed and has been applied to study plant growth, environmental interactions, and metabolism, among others. In recent years, transcriptomics and metabonomics have been used to explore the defence mechanisms of plant diseases and insect pests, such as tea, maize, and sesame [4–6], providing a new idea and method to explore the mechanism of resistance of roses to *Botrytis c.* infection.

Plants have complex defence mechanisms against *Botrytis c.*, including the MAPK cascade reaction, plant hormone signal transduction pathway, and cAMP signal pathway [7]. Silencing the MAPK-WRKY transcription factor gene increased the susceptibility of tobacco to *Botrytis c.*, indicating that the MAPK cascade was involved in the immune response of tobacco to fungi [8]. MPK3 and MPK6 can enhance the resistance of Arabidopsis to *Botrytis c.* by inducing the expression of the *glip1* gene [9]. Lu et al. identified *bZIP* transcription factors from the strawberry whole genome and found that the expression of *FvbZIP46* was significantly increased by *Botrytis c.* Transient overexpression and gene silencing of *FvbZIP46* indicated that *FvbZIP46* was involved in strawberry defence against *Botrytis c* [10]. Plant hormone signalling and transcription factors play an important role in plant resistance to *Botrytis c.* infection. Hu et al. found that exogenous application of N-decanoyl-homoserine lactone enhanced tomato resistance to *Botrytis c.* by activating JA biosynthesis and signal transduction in tomato [11]. Liu et al. found that BR signal transduction pathway-related genes were upregulated after infection with *Botrytis c.*, and exogenous BR enhanced its defence response to *Botrytis c.*, indicating that BR was involved in the resistance of rose to *Botrytis c* [12].. Exogenous 2,4-Epibrassinolide improves the resistance of grapes to *Botrytis c.* by inhibiting spore germination of *Botrytis c.* and enhancing the sensitization mechanism of grape fruit [13, 14]. In addition, some microRNAs are also involved in plant defence against *Botrytis c.* Nie et al. found that overexpressed miR825 and miR825 * improved the susceptibility of Arabidopsis to *Botrytis c.* In contrast, silencing miR825 and miR825 * enhanced the resistance of Arabidopsis to *Botrytis c.*, indicating that mir825 and mir825 * play a negative regulatory role in Arabidopsis defence against *Botrytis c* [15].

To explore the defence mechanism of rose against *Botrytis c.*, Old Blush was used as the experimental material, and the whole genome was published in 2018 (https://lipm-browsers.toulouse.inra.fr/pub/RchiOBHm-V2/). In this study, we measured the antioxidant enzyme activities of plants infected with *Botrytis c*. We then combined transcriptomic and metabolomic analyses to compare the gene expression and metabolomic profiles between CK and different infection time points (T36, T60, T72). These data provide significant information for the future breeding of rose resistance to grey mould.

## Results

### Effects of *Botrytis c.* on the phenotypic and biochemistry of old blush

**Fig. 1:**
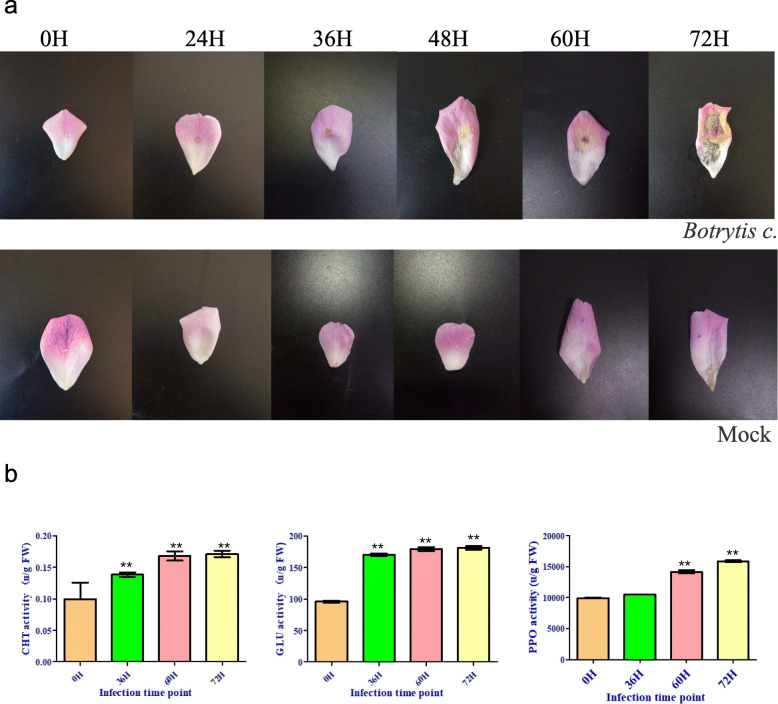
Changes in phenotype (**a**) after Old Blush was inoculated with sterile distilled water (SDW, mock) or *Botrytis c.* (**b**) defense enzyme activities. All statistical analyses were performed using Student’s t-test; *, *P* < 0.05; **, *P* < 0.01; hpi hours post-inoculation

We observed and recorded the phenotypic changes in Old Blush to investigate the effect of *Botrytis c.* infection on Old Blush growth. After 24 h of inoculation, the petals developed typical disease spots, which continued to grow larger to form apparent necrotic symptoms after 72 h of inoculation. In contrast, this pathogenic response was not observed in control petals treated with the mock control (Fig. 1a). During long-term evolution, plants have developed sophisticated biochemical and physiological mechanisms to adapt and resist various pathogenic bacteria. PPO, Glu and CHT play an important role in plant resistance to microbial infection. Therefore, enzymatic assays were performed to examine whether PPO, GLU and CHT were responsive to *Botrytis c.* infection. The results showed that the activities of these enzymes significantly differed among the treatments. The CHT activities of the 36 h, 60 h, 72 h petals increased by 26.13, 51.35 and 55.86%, respectively; the PPO activities at 36 h, 60 h, 72 h increased by 5.72, 41.87 and 59.66%, respectively; the GLU activities at 36 h, 60 h, 72 h increased by 78.65, 86.86 and 88.92%, respectively (Fig. 1b). These data indicate that the defence system in Old Blush is responsive to *Botrytis c.* infection.

### Overview of Transcriptomic analysis

**Fig. 2:**
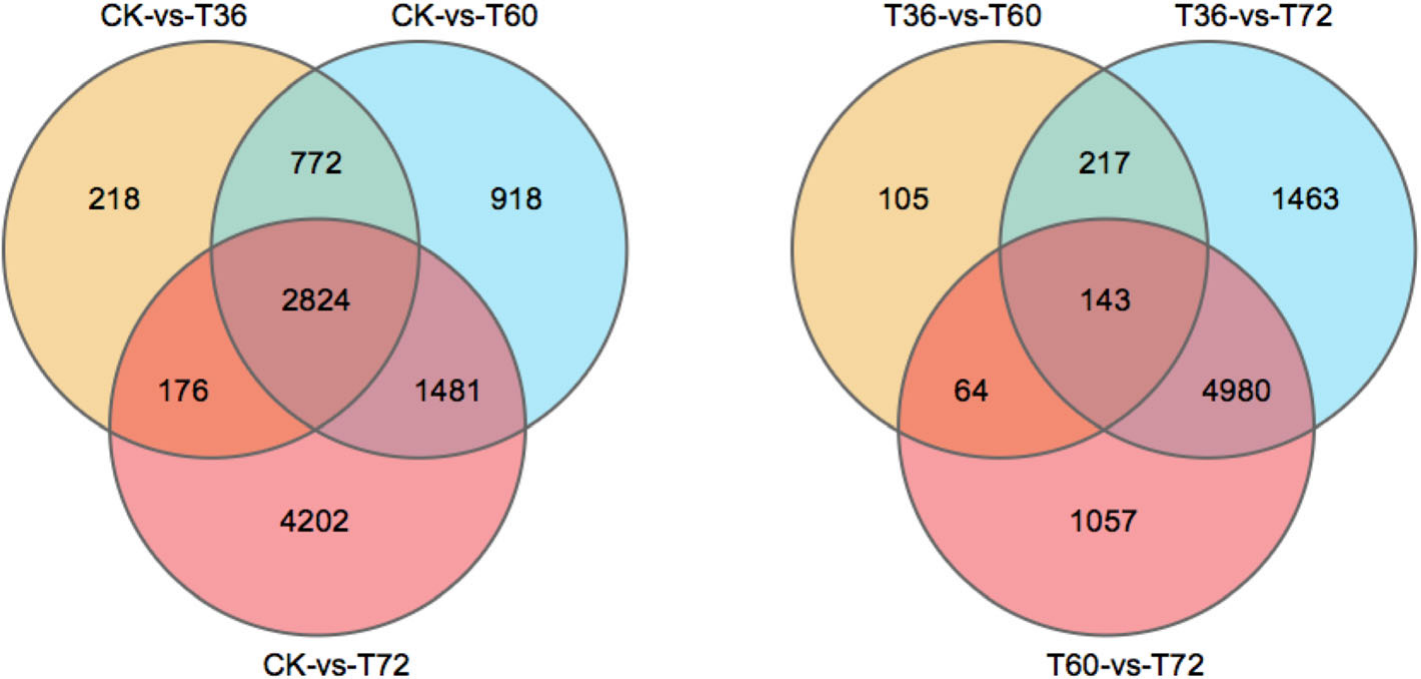
Venn map of differentially expressed genes. (CK) non-infected rose flowers, (T36) *Botrytis c.* inoculated rose flowers at 36 h, (T60) *Botrytis c.* inoculated rose flowers at 60 h, (T72) *Botrytis c.* inoculated rose flowers at 72 h

To understand the difference in gene expression between *Botrytis c.*-infected petals and mock petals, we next conducted an RNA-Seq analysis. A total of approximately 54 GB clean reads were generated from twelve biological samples, including nine infected and three control samples. The average Q20 value of the raw reads was 95.88%, indicating high-quality reads. Approximately 82% of the reads were mapped to the reference genome sequences obtained by Trinity splicing. To obtain a comprehensive view of the gene expression profile associated with the response of Old Blush to *Botrytis c.* infection, we used DESeq2 to identify DEGs. Based on the filtering parameters of FDR < 0.05 and |log2FC| > 1, the expressions of 3990 (2349 upregulated, 1641 downregulated), 5995 (3621 upregulated, 2374 downregulated) and 8683 (4164 upregulated, 4518 downregulated) genes were found to differ significantly in CK-VS-T36, CK-VS-T60 and CK-VS-T72, respectively. In addition, 529, 6803 and 6244 DEGs were identified in the comparison of T36 vs. T60, T60 vs. T72 and T36 vs. T72 (Fig. 2).

**Fig. 3:**
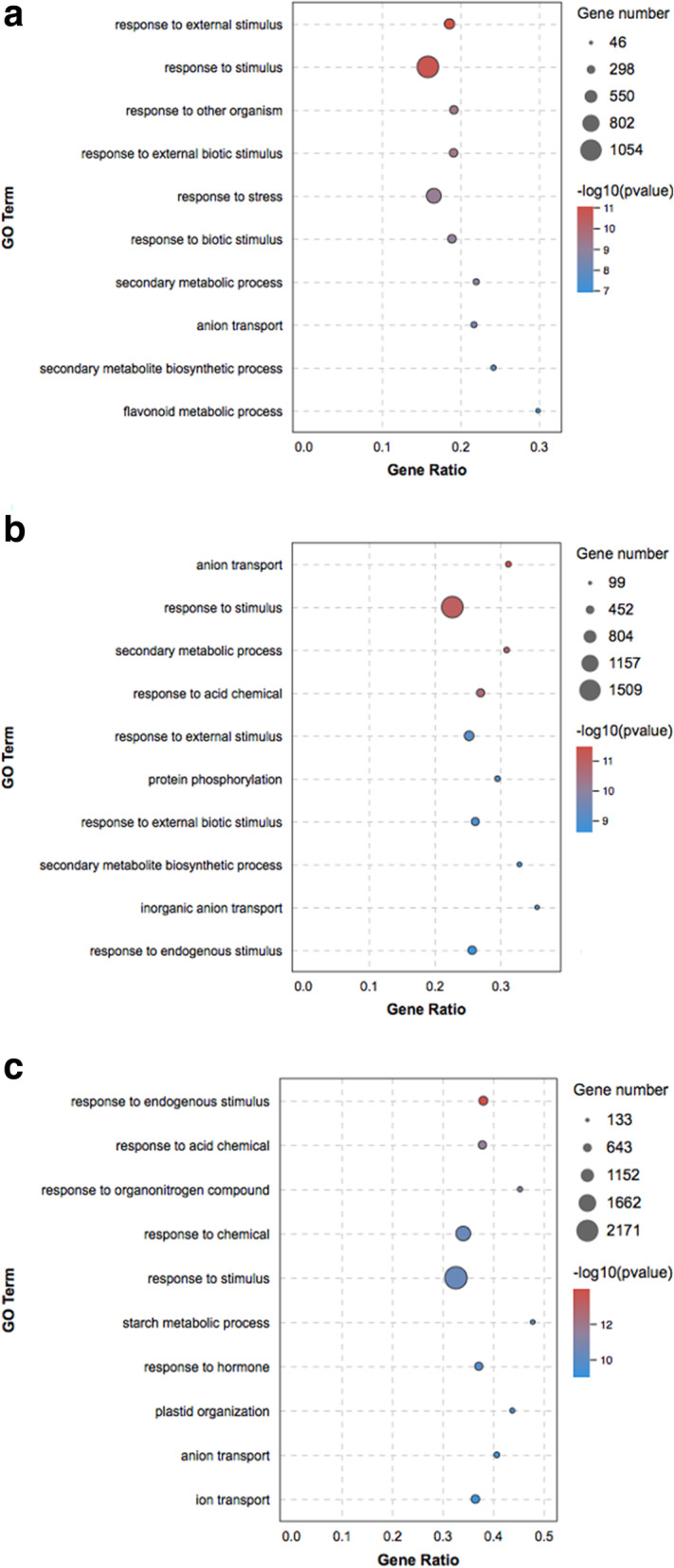
GO analysis based on DEGs in CK VS T36 (**a**), CK VS T60 (**b**) and CK VS T72 (**c**). (CK) non-infected rose flowers, (T36) *Botrytis c.* inoculated rose flowers at 36 h, (T60) *Botrytis c.* inoculated rose flowers at 60 h, (T72) *Botrytis c.* inoculated rose flowers at 72 h

To understand the functions of the DEGs associated with *Botrytis c.* infection, those DEGs were annotated using GOseq. This annotation resulted in three major categories: biological processes, cellular components, and molecular functions. Most of the DEGs were enriched in the ‘response to external stimulus’, ‘response to stimulus’, ‘response to external biotic stimulus’, ‘response to stress’, ‘secondary metabolic process’, ‘flavonoid metabolic process’ and other functional categories (Fig. 3a, b, c). The results showed that *Botrytis c.* infection activated rose resistance.

**Fig. 4:**
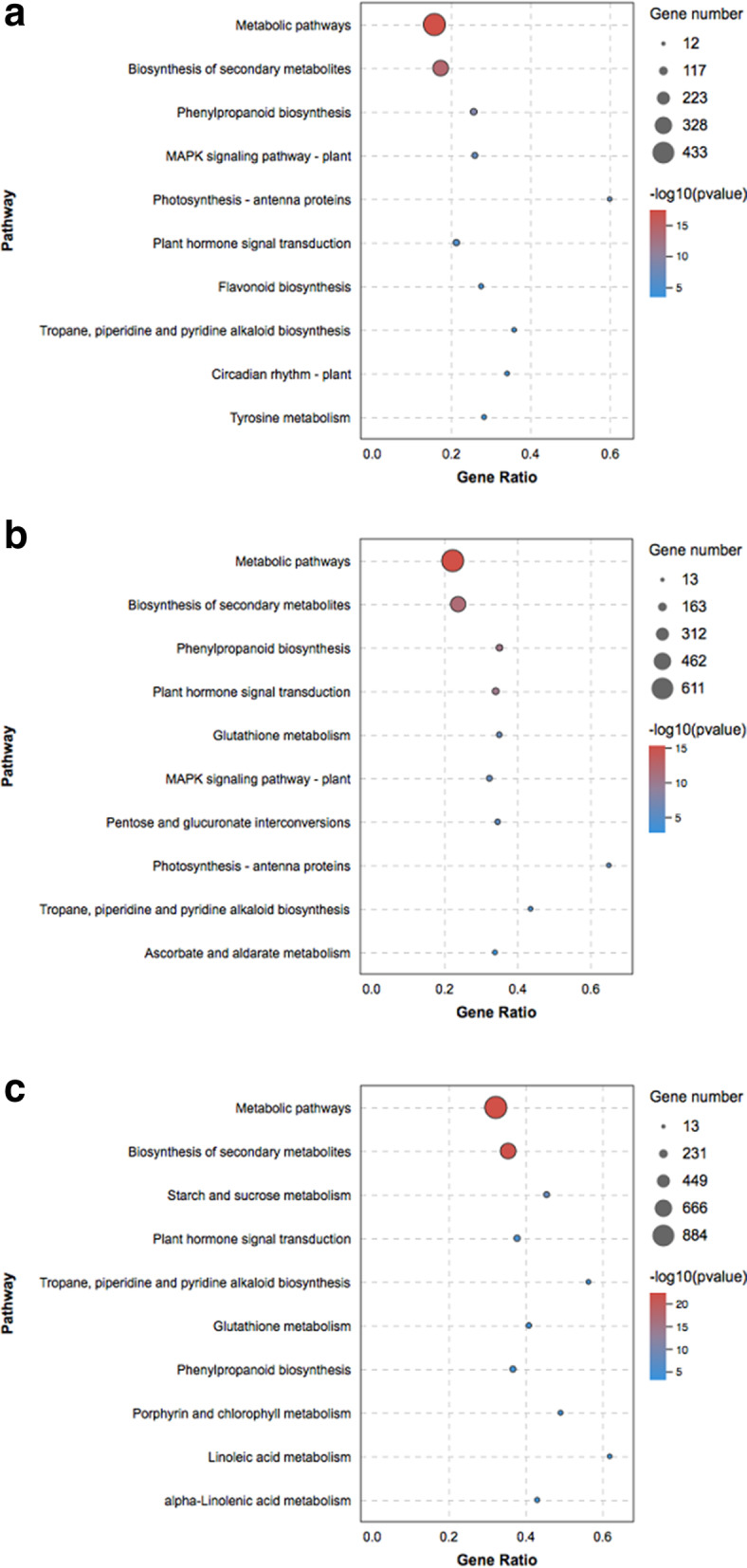
KEGG analysis based on DEGs in CK VS T36 (**a**), CK VS T60 (**b**) and CK VS T72 (**c**). (CK) non-infected rose flowers, (T36) *Botrytis c.* inoculated rose flowers at 36 h, (T60) *Botrytis c.* inoculated rose flowers at 60 h, (T72) *Botrytis c.* inoculated rose flowers at 72 h

To better understand the main pathways activated by *Botrytis c.* infection, we conducted a Kyoto Encyclopedia of Genes and Genomes (KEGG) enrichment analysis of the DEGs. Between the CK and T36 libraries, 728 DEGs were assigned to 127 KEGG pathways. Between the CK and T60 libraries, 1102 DEGs were assigned to 130 KEGG pathways. Between the CK and T72 libraries, 1607 DEGs were assigned to 134 KEGG pathways. Among these DEGs, genes involved in ‘metabolic pathways’ were the most abundant, followed by genes involved in ‘biosynthesis of secondary metabolites’. There were some important disease resistance pathways involved in the ‘MAPK signalling pathway – plant’, ‘phenylpropanoid biosynthesis’, ‘plant hormone signal transduction’, and ‘glutathione metabolism’ (Fig. 4a, b, c). This result indicated that a series of resistance pathways were activated in rose after infection with *Botrytis c.*

**Fig. 5:**
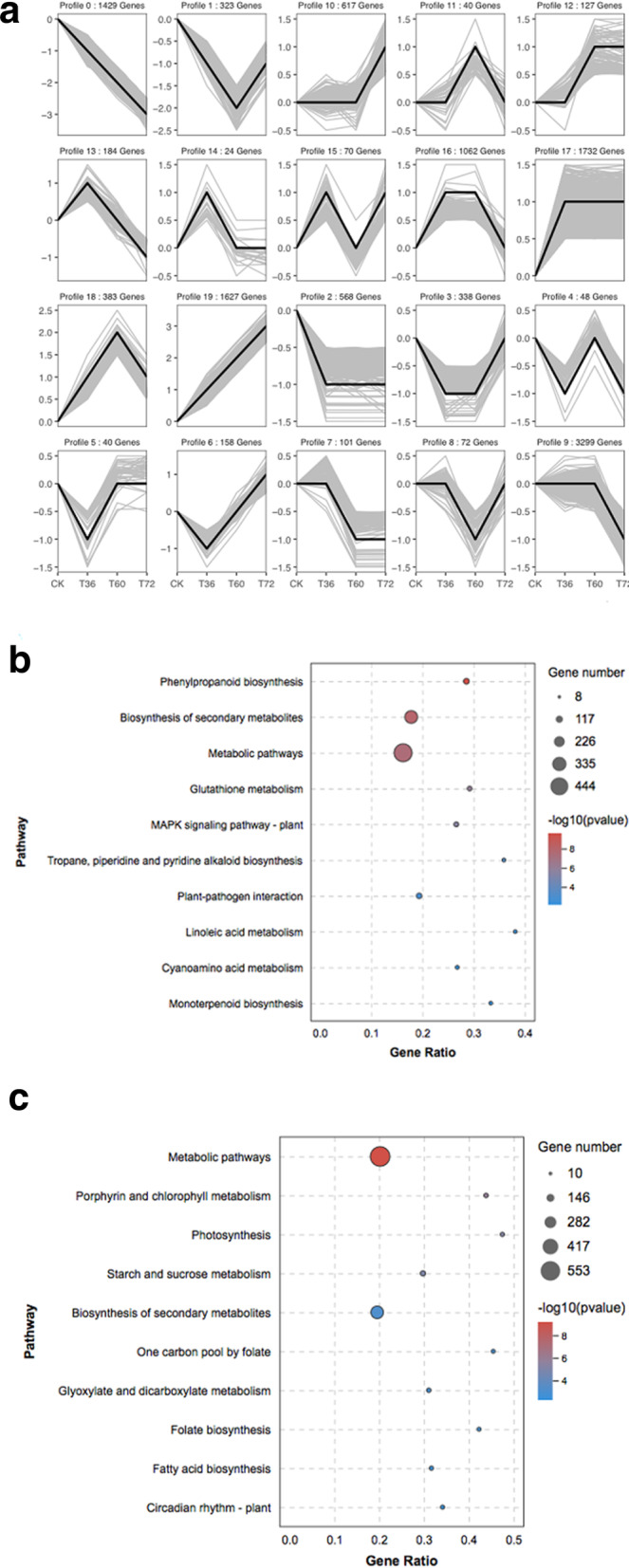
Analysis of DEGs. **a** Cluster analysis of DEGs; (**b**) KEGG enrichment Bubble Diagrams of profile 17 and profile 19 upregulated DEGs. **c** KEGG enrichment Bubble Diagrams of profile 0 and profile 9 downregulated DEGs

To understand the dynamics of differential gene expression by *Botrytis c.* infection, 12,842 DEG clustering analyses of expression patterns in different periods were performed by the Short Time-series Expression Miner. Through this analysis, the expression dynamics of all the DEGs at four periods could be clustered into 20 expression patterns, among which 10,334 DEGs showed 7 significant clustering patterns (*P* < 0.05). Compared with 0 h, the whole infection process included three downregulated expression patterns (modes 0, 9, 2) and four upregulated expression patterns (modes 19, 16, 10, 17). Among them, the number of downregulated genes in modes 9 and 0 was greater than that in mode 3. The number of upregulated genes in modes 17 and 19 was greater than that in modes 16 and 10 (Fig. 5a).

The downregulated genes with the most highly represented pathways were ‘metabolic pathways’ and ‘biosynthesis of secondary metabolites’. This also includes ‘starch and sucrose metabolism’, ‘porphyrin and chlorophyll metabolism’, ‘photosynthesis’, ‘glyoxylate and dicarboxylate metabolism’, ‘fatty acid biosynthesis’ and other plant development processes (Fig. 5c). These metabolic pathways are mainly involved in primary metabolism in plants, indicating that the expression of genes involved in primary metabolism decreased and the process of primary metabolism weakened after infection with *Botrytis c.*

In contrast, the upregulated genes were the most highly represented pathways in addition to ‘metabolic pathways’, ‘biosynthesis of secondary metabolites’, ‘phenylpropanoid biosynthesis’, ‘plant pathogen interaction’, ‘MAPK signalling pathway-plant’, ‘plant hormone signal transduction’, ‘alpha-linolenic acid metabolism’, ‘glutathione metabolism’ and ‘monoterpenoid biosynthesis’ (Fig. 5b). These metabolic pathways are mainly involved in the secondary metabolism of plants, indicating that the expression of genes involved in secondary metabolite synthesis is increased to enhance the resistance of rose to *Botrytis c.* Taken together, the statistical results above indicate that rose starts its own defence mechanism in response to *Botrytis c.* stress by balancing primary and secondary metabolism.

**Fig. 6:**
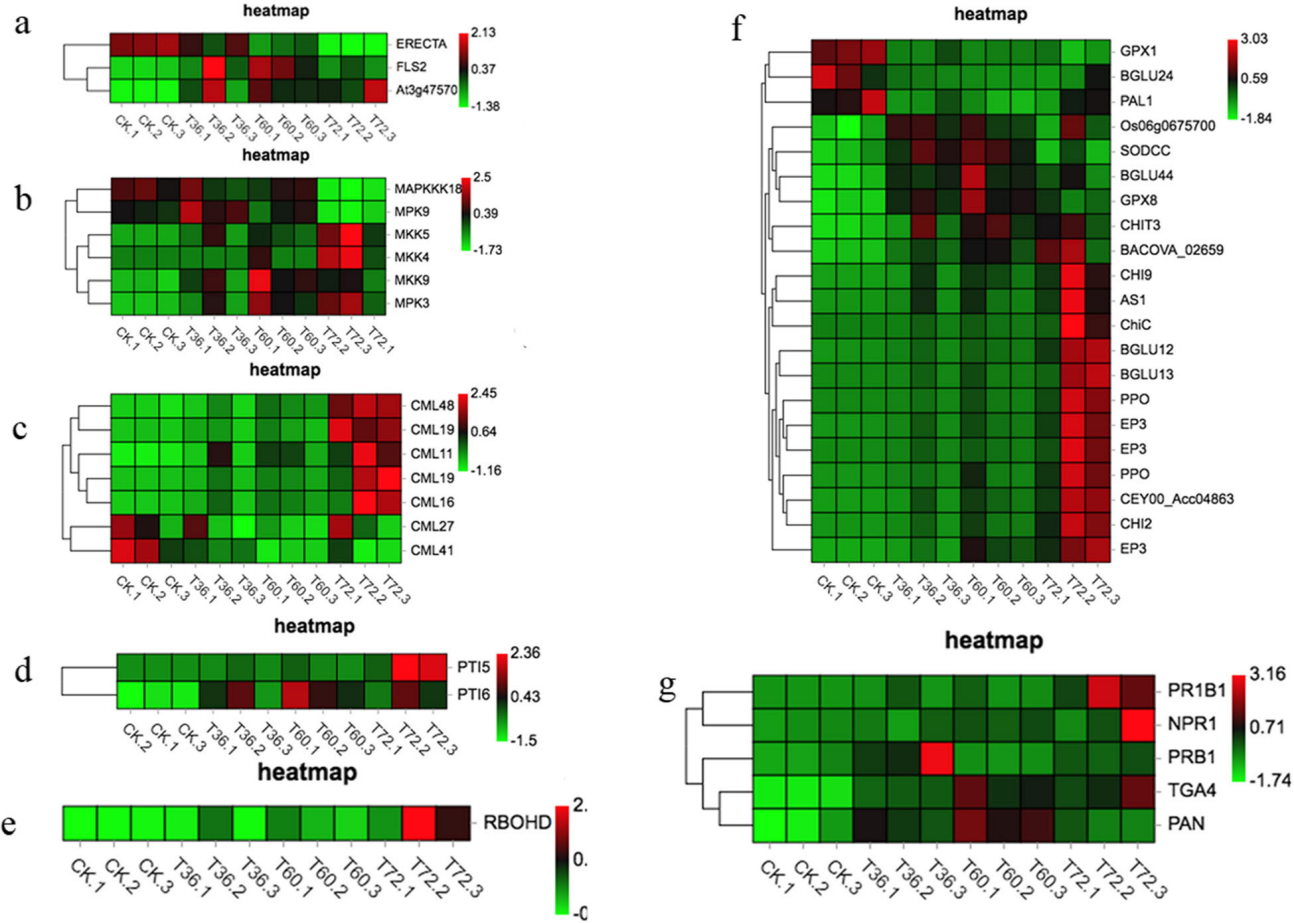
Heatmap of genes in Old Blush in response to *Botrytis c.* infection. The bar represents the scale of the expression levels for each gene (FPKM) in the different treatments, as indicated by red/green rectangles. Genes in red show upregulation, and those in green show downregulation. **a** R genes, **b** MAPK signaling pathway genes, **c** Ca^2+^ signaling pathway, **d** PTI genes, **e** ROS metabolic pathway, **f** Defense enzyme, **g** SA signaling pathway

Heatmaps of DEG subclusters were developed to better understand the key DEGs associated with the resistance of Old Blush to *Botrytis c.* The resulting heatmaps showed DEGs involved in plant-pathogen interactions. Based on their functional annotation, these genes included 2 PAMP-triggered immunity (PTI) genes, 3 R genes, 1 reactive oxygen species (ROS) metabolic pathway gene, 4 calmodulin genes, 3 calcium-binding protein genes, 6 mitogen-activated protein kinase (MAPK) signalling pathway genes, and 5 DEGs involved in the SA response pathway, including 1 NPR gene, 2 PRs, and 2 transcription factor TGA genes. In addition, 21 defence enzyme genes, including one PAL, 8 GLUs, 7 CHTs, 2 PPOs, 2 GPXs and one SOD unigene, are shown in the heatmap (Fig. 6). This indicated the rose response to *Botrytis c.* by activating the expression of signal transduction pathway genes, transcription factors and disease resistance genes.

### Validation of candidate DEGs with qPCR analysis

**Fig. 7:**
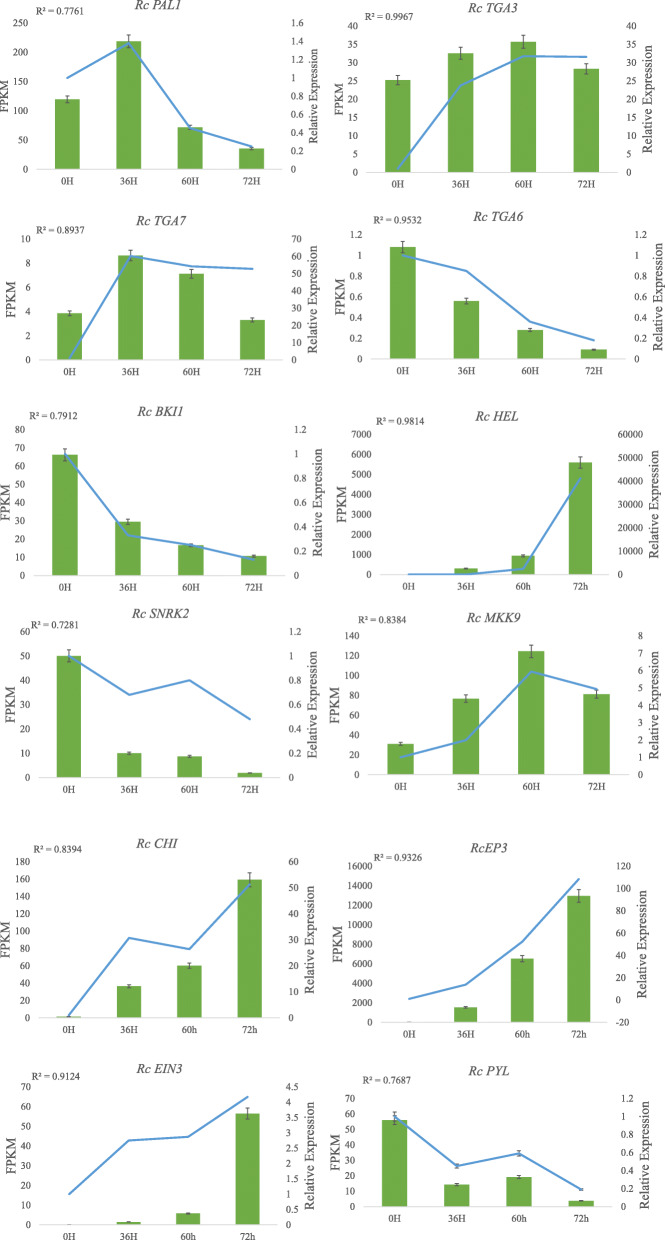
The expression levels of 12 DEGs identified in Old Blush in association with the response to *Botrytis c.* infection. The correlations between the expression profiles of the 12 DEGs were determined by RNA-Seq and qPCR analysis. The left y-axes show FPKM values determined by RNA-Seq, and the right y-axes show relative expression levels determined by qPCR. *Rc UBC* was used as a reference gene. The r value between the RNA-Seq and qPCR results is listed in the left corner of each figure representing gene expression

To validate the reliability of DEGs obtained from RNA-Seq analyses, the expression levels of 12 candidate genes were analysed using qPCR. These genes included 3 TGAs (*RcTGA3/6/7*), 4 defence enzyme genes (*RcPAL1, RcPYL, RcCHI,* and *RcEP3*), 2 plant hormone signalling pathway genes (*RcEIN3 and RcBKI1*), one pathogenesis-related gene (*RcHEL*), and 2 MAPK signalling pathway genes (*RcMKK9* and *RcSNRK2*). The correlation coefficients (r) between the RNA-Seq and qPCR results were calculated for these DEGs (Fig. 7). The results showed that the correlation coefficients were greater than 0.7, indicating that the RNA-Seq data were reliable.

### Silencing of *RcTGA1* enhances susceptibility to *Botrytis c*

**Fig. 8:**
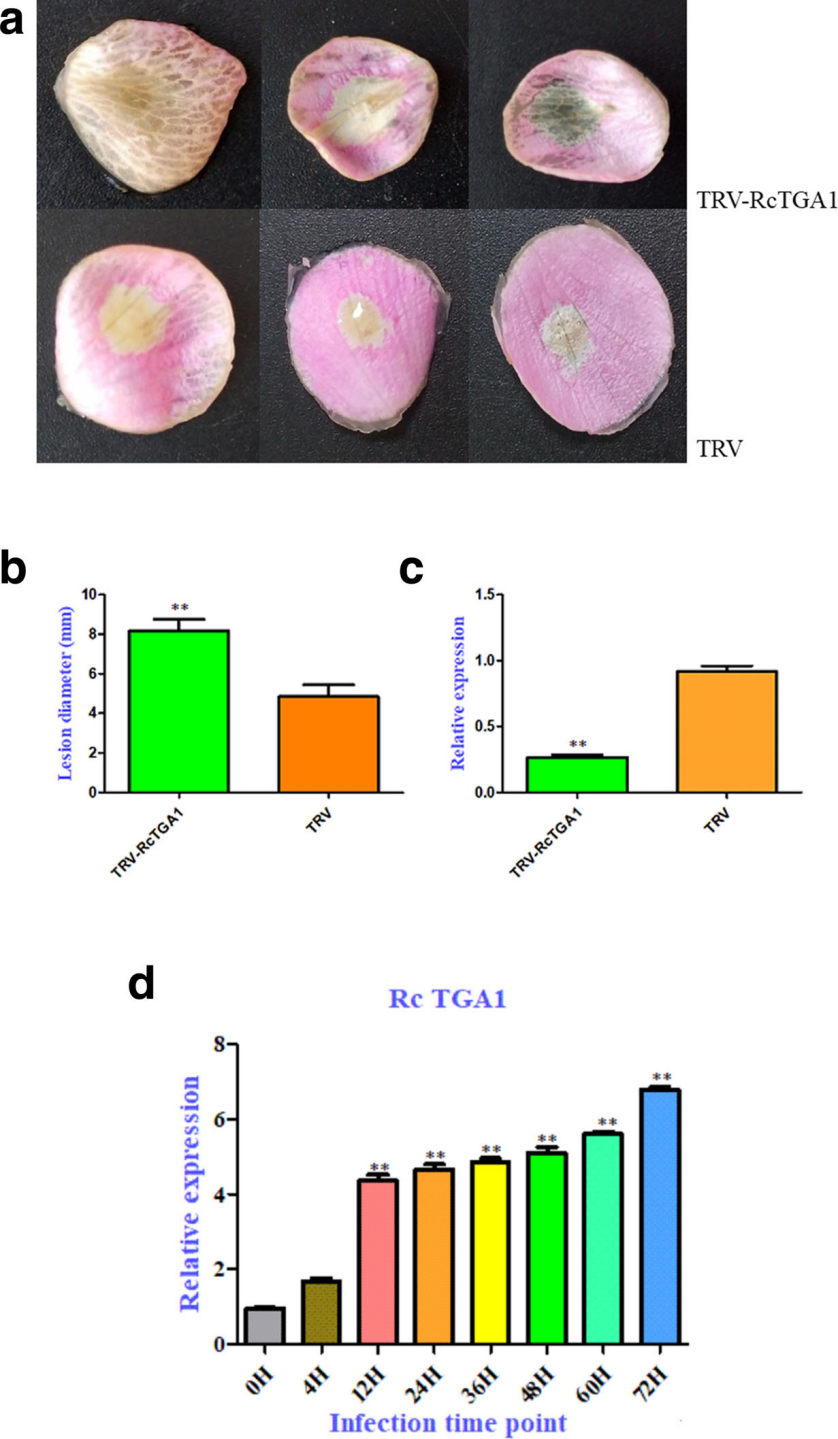
*Botrytis c.* inoculation of rose petal discs after virus-induced gene silencing of *RcTGA1* (**a**) Petal discs from the Old Blush was inoculated with empty tobacco rattle virus (TRV) as a control (TRV) or with a recombinant TRV targeting *RcTGA1* (TRV-RcTGA1). Compromised resistance to *Botrytis c.* was observed in *RcTGA1*-silenced plants at 48 h. **b** Compromised *Botrytis c.* resistance upon silencing of *RcTGA1*. **c** Quantification of *RcTGA1* expression in TRV-*RcTGA1*-inoculated petal discs relative to that in the control. **d** the expression characteristics of *RcTGA1* after inoculation with *Botrytis c.* Statistical analysis was performed using Student’s t-test; *, *P* < 0.05; **, *P* < 0.01

TGA1-TGA7 can interact with NPR1, a key regulator of the salicylic acid signalling pathway, to regulate plant disease resistance in *Arabidopsis thaliana*. Based on NCBI BLASTx, we identified and isolated the *RcTGA1* (gene ID: XM024330029.1) gene located in the SA resistance pathway. To detect the expression pattern of *RcTGA1* in response to *Botrytis c.*, we inoculated *Botrytis c.* on the flowers of Old Blush and detected the expression characteristics of *RcTGA1* in flowers using qPCR (Fig. 8d). The results showed that the expression level of *RcTGA1* increased significantly after 4 h of infection. It continued to grow within 72 h after infection with *Botrytis c.* This result suggested that *RcTGA1* might be involved in the response of rose to *Botrytis c.*

It has been previously demonstrated that TRV-mediated VIGS can be used in rose as a tool for the functional analysis of genes involved in flower development and the determination of petal colour and fragrance [16, 17]. To determine whether the increased expression of genes of the SA response pathway is associated with the tolerance of Old Blush to *Botrytis c.*, we cloned fragments of *RcTGA1* (an upregulated DEG encoding a transcription cofactor related to the SA signalling pathway) that were 250 bp in length into the TRV vector and inoculated rose petal discs with these fragments via vacuum infiltration. At one-week post-infiltration, we subsequently challenged TRV-RcTGA1- and TRV*-*inoculated rose petal discs with *Botrytis c.* The TRV-RcTGA1-inoculated plants showed severely compromised resistance, as evidenced by significantly increased lesion size (Fig. 8a, b). We further used qPCR to detect the silencing efficiency in the petals. The results showed the most significant decrease in *RcTGA1* expression upon infiltration with TRV-RcTGA1 (Fig. 8c). These results indicated that *RcTGA1* is essential for *Botrytis c.* resistance in rose.

### Overview of Metabonomic analysis

To understand the changes in metabolites and the possible defence mechanisms of Old Blush infected by *Botrytis c.*, metabolite profiling analysis of Old Blush flower samples (CK, T36, T60, T72) was performed. A total of 635 metabolites were detected in all samples, which could be divided into 29 groups (Table S1). Principal component analysis (PCA) showed that the repeatability of different treatments was good. Orthogonal partial least squares discriminant analysis (OPLS-DA) showed that the results of OPLS-DA analysis could be used for subsequent model tests and differential metabolite analysis. Between the CK and T36 treatments, 373 were upregulated, and 262 were downregulated. Between the CK and T60 treatments, 397 were upregulated, and 238 were downregulated. Between the CK and T72 treatments, 406 were upregulated, and 229 were downregulated. The levels of piperidine, γ-aminobutyric acid, L-histidine, 5-O-p-coumaroylquinic acid, chlorogenic acid, terminal acid, 2-hydroxyoleanolic acid, 2α-hydroxyursolic acid, and 3,4-digalloylshikimic acid increased significantly with the extension of infection time. This indicated that these secondary metabolites were involved in the resistance response of rose to *Botrytis c.*

To identify the main pathways that Old Blush uses to respond to *Botrytis c.,* we mapped the differentially expressed metabolites to KEGG biological pathways. Thirty-two significantly differentially expressed metabolites between the CK and T36 treatments were assigned to 50 KEGG pathways, including ‘aminoacyl-tRNA biosynthesis’, ‘valine, leucine and isoleucine biosynthesis’, and ‘biosynthesis of amino acids’. Thirty-eight significantly differentially expressed metabolites between the CK and T60 treatments were assigned to 61 KEGG pathways, including ‘aminoacyl-tRNA biosynthesis’, ‘2-oxocarboxylic acid metabolism’, and ‘glucosinolate biosynthesis’. Thirty-seven significantly differentially expressed metabolites between the CK and T72 treatments were assigned to 65 KEGG pathways, including ‘biosynthesis of antibiotics’, ‘valine, leucine and isoleucine biosynthesis’, ‘alanine, aspartate’ and ‘glutamate metabolism’ (Table S2). The results showed that the metabolic pathways related to disease resistance were significantly enriched, indicating that the defence mechanism of rose was activated after infection by *Botrytis c.*

### Glucosinolate biosynthesis

The glucosinolate biosynthesis pathway is involved in the defence responses of many plants to pathogens. In our research, the key metabolites of the glucosinolate biosynthesis pathway, including L-valine, L-isoleucine, and L-leucine, exhibited different levels in different treatments. Compared with that in the CK, their level in T36 increased by factors of 1.78, 1.09, and 1.08, respectively; in T60, they increased by factors of 2, 2.3, and 2.4, respectively, and in T72, they increased by factors of 1.6, 2.0, and 2.0, respectively. These results indicate that the glucosinolate biosynthesis pathway may be positively involved in the interaction between rose and *Botrytis c.*

### Co-joint analysis

**Fig. 9:**
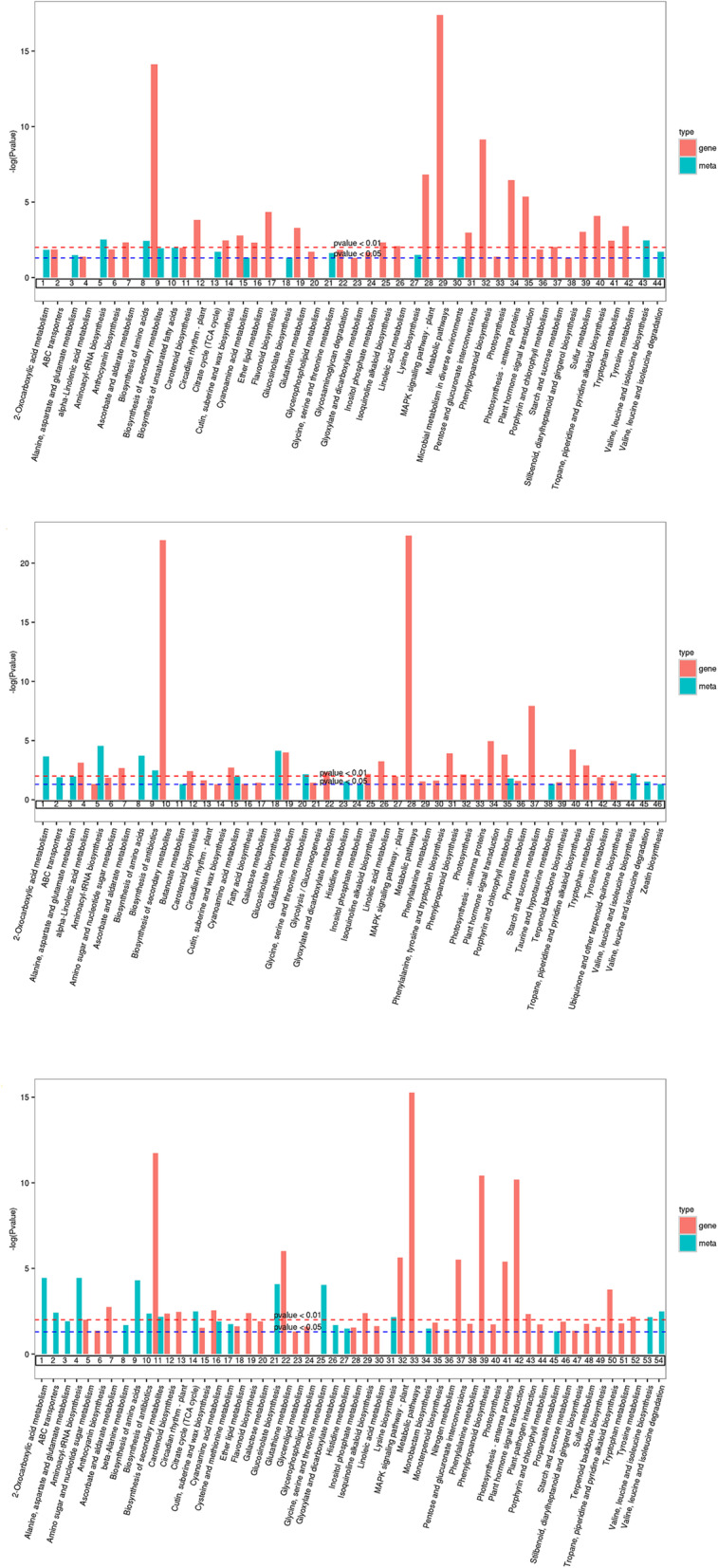
Joint KEGG enrichment *p*-value histogram in CK VS T36 (**a**), CK VS T60 (**b**) and CK VS T72 (**c**). (CK) non-infected rose flowers, (T36) *Botrytis c.* inoculated rose flowers at 36 h, (T60) *Botrytis c.* inoculated rose flowers at 60 h, (T72) *Botrytis c.* inoculated rose flowers at 72 h

The co-joint KEGG enrichment analysis showed 85 co-mapped pathways. There were 46, 56, and 60 comapping pathways between CK-VS-T36, CK-VS-T60, and CK-VS-T72 and their metabolites. Interestingly, of these co-mapped pathways, ‘metabolic pathways’, ‘biosynthesis of secondary metabolites’, ‘phenylpropanoid biosynthesis’, ‘flavonoid biosynthesis’, ‘tropane, piperidine and pyridine alkaloid biosynthesis’, ‘tyrosine metabolism’, ‘pentose and glucuronate interconversions’, ‘cyanoamino acid metabolism’, and ‘ascorbate and aldarate metabolism’ were their common pathways of significant enrichment (Fig. 9a, b, c). This indicates that rose responds to pathogen infection by coordinating the primary and secondary metabolic pathways. Based on the O2PLS model, the combined analysis of transcriptomics and metabonomic data showed that the model was reliable (R2 > 0.85). The Pearson correlation coefficients showed that the differential expression patterns of DEGs and metabolites were consistent. The correlations between the top 250 DEGs and their metabolites were further selected and are represented as a heat map (Fig. s1).

## Discussion

### RNA-seq study for disease resistance

As an important economic horticultural plant, rose infected with *Botrytis c.* during its growth or after harvest will cause great economic losses to farmers [18]. To understand the mechanism of Old Blush tolerance to *Botrytis c.*, RNA-Seq and metabolomics were employed to analyses DEGs and DEMs in Old Blush at different time points after inoculation with *Botrytis c.* In our study, a large number of DEGs and DEMs were putatively related to the response of Old Blush to necrotrophic *Botrytis c.* Numerous DEGs are involved in various defense responses to *Botrytis c.*, including redox-related genes, Ca^2+^, MAPK signalling pathway-related genes and plant hormone signal transduction pathway-related genes. All of these transcriptomic data indicate that multiple processes in Old Blush are associated with plant defence against pathogens, which is in line with the fact that the plants have evolved a complex defence mechanism [19–22]. When infected by pathogens, the plant recognizes the PAMP secreted by *Botrytis c.* through the pattern recognition receptor on the cell surface and triggers a series of cellular reactions, including ROS production, changes in cytoplasmic ion flux, calcium-dependent protein and MAPK cascade activation. In the process of preventing pathogen infection, plants encode the R protein to activate effector-triggered immunity and produce a hypersensitive reaction (HR) at the infected site, which promotes cell death [23]. In our study, FLS2 and ROS pathway genes were significantly upregulated after *Botrytis c.* infection. This result revealed that *Botrytis c.* infection also triggered effector-triggered immunity of Old Blush.

### Resistance of JA and SA signalling pathways to *Botrytis c.*

Plant hormones, such as salicylic acid and jasmonic acid, are involved in the plant defence response to plant diseases and insect pests [24, 25]. Ren et al. silenced the key JA synthesis gene AOS and JA coreceptor gene COI1, which increased the susceptibility of Old Blush to *Botrytis c.*, indicating that the JA pathway plays an important role in resisting *Botrytis c.* to rose [12]. Liu et al. analysed transcriptome data and found that BR hormone signal transduction pathway-related genes were upregulated after rose inoculation. Exogenous BR enhanced the resistance of rose petals to *Botrytis c.* In this study, Old Blush was inoculated with *Botrytis c.,* a typical necrotrophic pathogen. The analysis of DEGs revealed the upregulation of JA signalling pathway genes, including *RcJAZ*, *RcLOXs,* and *RcCOI*. DEM analysis showed that the content of α-linolenic acid* related to JA synthesis also increased significantly, which was consistent with the results of Ren. SA activates NPR1 expression causing *Arabidopsis* to gain systemic resistance and improve its disease resistance. SA application to tomato leaves significantly increased the expression level of the SA marker gene PR1 (pathogenesis-related protein 1) and enhanced its resistance to *Botrytis c.* In our study, the expression of the SA pathway genes *RcTGAs* and PR1s was upregulated in Old Blush after inoculation with *Botrytis c.* This result suggested that SA may be involved in the resistance of Old Blush to *Botrytis c.* in the early stage.

### Functional verification of *RcTGA1*

Tianyi et al. [26] found that overexpression of MdTGA2.1 can complement the SA-sensitive phenotype of TGA2/5/6 in *Arabidopsis thaliana*. Van Verk et al. [27] showed that TGA2.2 could specifically interact with NtWRKY12 and regulate the expression of PR-1a in vivo and in vitro. All of these studies indicate that TGAs are broad-spectrum resistance genes in the salicylic acid resistance pathway. In our study, the expression of *RcTGA1* continued to increase significantly after infection with *Botrytis c.* RcTGA1 was silenced in rose petals and then inoculated with *Botrytis c.*, and the lesion diameter on the petal disc was twice that of the control. Taken together, *RcTGA1* positively regulates rose tolerance to *Botrytis c.*

### Metabolomics study for disease resistance

Secondary metabolites determine the colour, smell and taste of plants. They are widely involved in plant growth, development, defence or other physiological processes. When plants are infected with pathogens, secondary metabolites are produced to participate in plant disease resistance [28]. Studies have shown that sugar metabolism, a primary metabolic process, affects plant susceptibility and plays a key role in innate defence pathways. The analysis of DEMs showed that compared with CK, the levels of saccharides and alcohols (D-threnodies, D-glucosamine, inositol, and D-glucose-6-phosphate) significantly decreased, which was consistent with the transcriptome data showing that the expression of genes related to the starch and sugar metabolism pathway was significantly downregulated. It is also known that alkaloids, triterpenoids, tannins and phenolic acids are involved in plant disease resistance. In this study, the content of secondary metabolites related to disease resistance increased, while the content of metabolites used for growth and development decreased. This result indicates that when infected by *Botrytis c.*, the roses increased their contents of disease-resistant metabolites and maintained their own growth and development simultaneously.

### Glucosinolate metabolism

Glucosinolates have broad antibacterial activity. Research has shown that the glucosinolate metabolism pathway is necessary for Arabidopsis immune pathogens [29]. Stotz et al. [30] showed that the content of glucosinolates in Arabidopsis increased and that the expression of glucosinolate synthesis-related genes was activated by *Sclerotinia sclerotiorum* infection. In our study, when rose was infected by pathogens, the expression of related genes and the content of metabolites increased significantly. Thus, the glucosinolate metabolic pathway may be involved in the resistance of Old Blush to *Botrytis c.*

### Regulation mechanism of resistance to *Botrytis c*

**Fig. 10:**
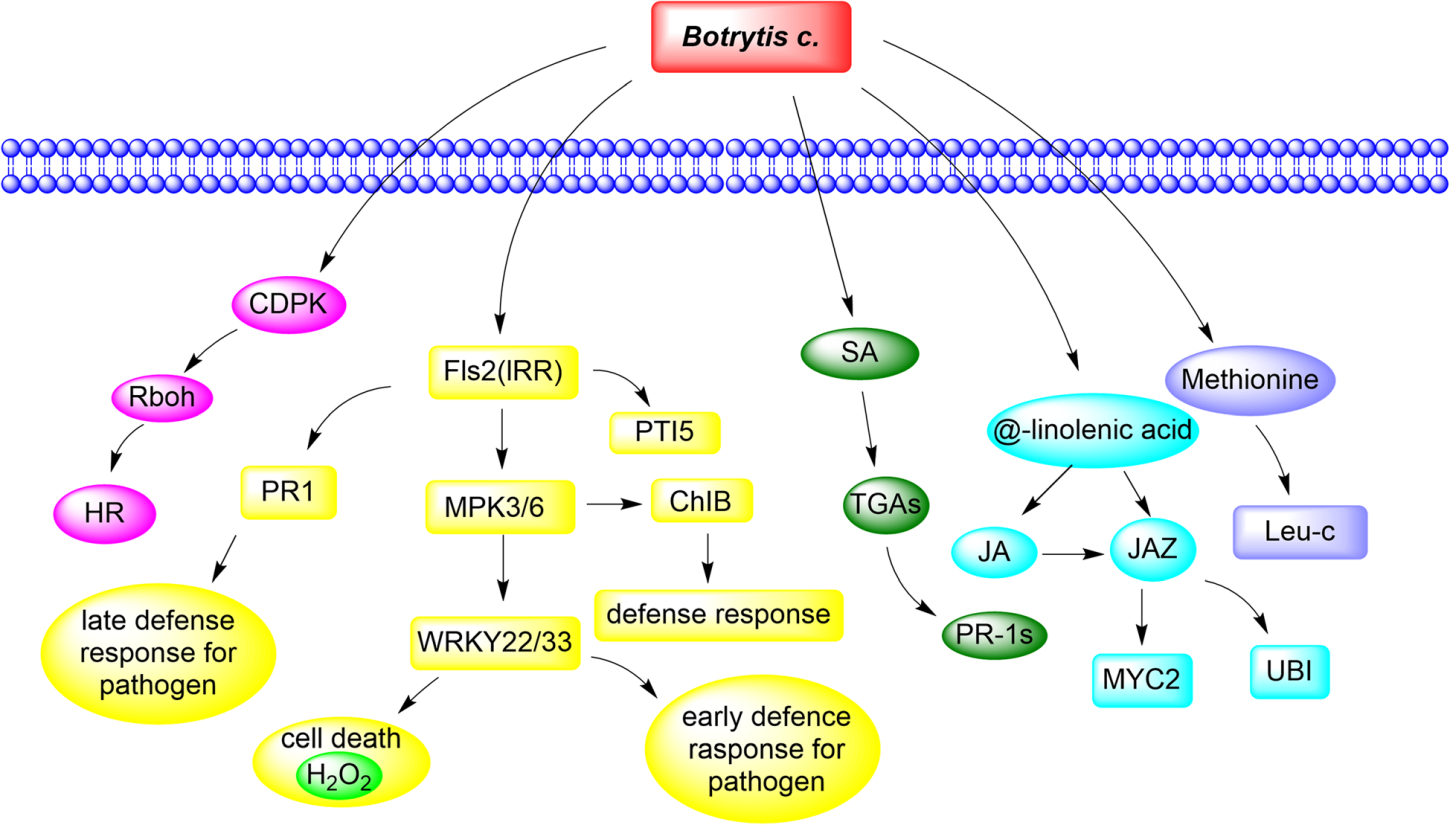
Hypothetical model of the mechanism of Old Blush tolerance to *Botrytis c*

Based on our work, we propose a hypothetical model to explain the resistance of Old Blush to *Botrytis c.* In this model, the plants recognize PAMPs secreted by fungi through their innate recognition receptors, causing a series of cellular responses. At the same time, the plant R protein activates ETI to induce plant hypersensitivity, which leads to cell death and prevents further infection. In addition, the expression of JA synthesis- and signal transduction pathway-related genes *RcJAZ* and *RcLOXs* is activated. The expression of SA-induced disease resistance-related genes, including *RcTGAs* and *RcPR1*s, also increased. The levels of precursors of JA synthesis alpha-linolenic acid and glucosinolate metabolic pathway metabolites, such as L-leucine and L-valine, increased significantly (Fig. 10). Taken together, we speculate that the MAPK-plant signalling pathway, CDPK gene, JA biosynthesis, SA resistance pathway and glucosinolate metabolism pathway are involved in the defence of rose against *Botrytis c.*

## Conclusion

In conclusion, 54 GB of clean reads were generated through RNA-Seq. A total of 3990, 5995 and 8683 DEGs were obtained in the comparisons of T36h, T60h and T72h, respectively, compared to CK. Functional annotation and cluster analysis of the DEGs showed that a variety of defence responses mediated by R proteins, ROS signalling, Ca^2+^ signalling, MAPK signalling, and SA signalling were activated in the Old Blush response to *Botrytis c. RcTGA1* positively regulates rose resistance to *Botrytis c*. Metabonomic data showed that a total of 59, 78 and 74 DEMs were obtained in T36h, T60h and T72h compared to CK, respectively. A variety of secondary metabolites are related to biological disease resistance, including tannins, amino acids and derivatives, and alkaloids, and they were significantly increased and enriched in phenylpropanoid biosynthesis, glucosinolates and other disease resistance pathways.

## Methods

### Plant growth and plant infection

*R. chinensis* Old Blush was grown at the rose germplasm garden of the Flower Research Institute, Yunnan Agriculture Academic Science, Kunming, China.

The *Botrytis c.* inoculum was produced by growing strain *B05.10* on solid medium (potato dextrose agar; 46 g^-1*L^ dH_2_O, pH ≈ 5.6) at 22 °C for 10 ~ 14 days. Spore inoculum was prepared by harvesting spores in water, filtering through glass wool to remove the hyphae, and suspending the filtrate in potato dextrose broth (PDB; 24 g per L dH_2_O) at 10^5^ conidia^-1*ml^ [31]. Four 2-μL drops of *Botrytis c.* inoculum or PDB (mock) were dropped onto each petal disk. Infected and control disks were individually sampled in a randomized manner from each of the three trays at 36 h, 60 h, and 72 h with three biological repeats for both infected and control treatments at each time point. Petals were immediately frozen in liquid nitrogen at the time of harvesting and stored at − 80 °C.

### Measurement of antioxidant enzyme activities

Frozen flower samples were used to determine the activity of the defense enzymes, including polyphenol oxidase (PPO), chitinase (CHT), and glucan endo-1,3-beta-glucosidase (GLU), and RNA-Seq was conducted. SA and JA contents were determined using an enzyme-linked immune sorbent assay (ELISA) in a facility at the Hanling BiO Company (Kunming, China). This company’s service helped to bind those enzymes (bovine serum albumin) and then produced the corresponding antibodies.

### RNA extraction, library construction and sequencing

Total RNA was extracted using a Trizol reagent kit (Invitrogen, Carlsbad, CA, USA) according to the manufacturer’s protocol. RNA quality was assessed using an Agilent 2100 Bioanalyzer (Agilent Technologies, Palo Alto, CA, USA) and checked using RNase-free agarose gel electrophoresis. After total RNA was extracted, eukaryotic mRNA was enriched by oligo(dT) beads, while prokaryotic mRNA was enriched by removing rRNA with a Ribo-Zero™ Magnetic Kit (Epicentre, Madison, WI, USA). Then, the enriched mRNA was fragmented into short fragments using a fragmentation buffer and reverse transcribed into cDNA with random primers. Second-strand cDNA was synthesized by DNA polymerase I, RNase H, dNTPs and buffer. Then, the cDNA fragments were purified using a Qia Quick PCR extraction kit (Qiagen, Venlo, The Netherlands), end repaired, poly(A) added, and ligated to Illumina sequencing adapters. The ligation products were size-selected by agarose gel electrophoresis, PCR-amplified, and sequenced using an Illumina HiSeq2500 [32].

### Transcriptomic data analysis

To obtain high-quality clean reads, we removed the adaptor-containing sequences, poly-N, and low-quality reads. The remaining clean reads were further used in the assembly and gene abundance calculation. Then, clean reads were mapped to the reference genome using the HISAT2 tool [33]. For each transcription region, an FPKM (fragment per kilobase of transcript per million mapped reads) value was calculated to quantify its expression abundance and variations using StringTie software [34, 35].

Differential expression analyses among the four treatments (CK vs. T36/60/72, T36 vs. T60, T36 vs. T72, and T60 vs. T72 with three biological replicates per treatment) were conducted using DESeq2 software [36]. Genes/transcripts with a false discovery rate (FDR) below 0.05 and absolute fold change of ≥2 was considered differentially expressed genes/transcripts.

GO enrichment analysis provided all GO terms that were significantly enriched in the DEGs compared to the genome background and filtered the DEGs that correspond to biological functions. KEGG [37] is the major public pathway-related database. Pathway enrichment analysis identified significantly enriched metabolic pathways or signal transduction pathways in the DEGs compared with the whole genome background.

### Quantitative real-time PCR validation

qPCR was used to validate the RNA-seq data for 12 different genes. Specific primers were designed using Premier 5 software (Premier Biosoft, Palo Alto, CA, USA). The RNA samples were used to synthesize cDNA, and a Step OnePlus Real-Time Fluorescent Quantitative PCR system (Trans Start® Green qPCR Super Mix) was used to monitor the amount of DNA. Assays of each gene were repeated three times. Quantification was evaluated using the 2^−(ΔΔCt)^ method.

### Functional verification of *RcTGA1*

To obtain the TRV-*RcTGA1* expression vector, a 250-bp fragment from the ORF of *RcTGA1* was cloned into the TRV vector PTRV2 and then electroporated into Agrobacterium strain GV3011. To establish VIGS in rose petals, detached petals were obtained from the outermost whorls of rose flowers at stage 2 of flower opening. Then, a 12-mm disc was punched from the centre of each petal. The petal discs were vacuum infiltrated with Agrobacterium carrying TRV constructs as described by Zhang and Thomma [38]. VIGS was repeated at least three times using at least 16 discs in each experiment. After *Botrytis c.* inoculation, Student’s t-test was conducted. All primers are listed in Supplemental Table S1.

### Extraction and quantification of metabolites

Metabolites were extracted from petals with five replicates per treatment. The compounds extracted were analysed using an LC-ESI-MS/MS system (UPLC, Shim-pack UFLC SHIMADZU CBM30A, http://www.shimadzu.com.cn/; MS/MS (Applied Biosystems 6500 QTRAP)). LIT and triple quadrupole (QQQ) scans were acquired on a triple quadrupole-linear ion trap mass spectrometer (Q TRAP) [39], an AB Sciex QTRAP6500 System, equipped with an ESI-Turbo Ion-Spray interface, operating in positive ion mode and controlled by Analyst 1.6.1 software (AB Sciex). The operation parameters were as follows: ESI source temperature 500 °C; ion spray voltage (IS) 5500 V; curtain gas (CUR) 25 psi; and collision-activated dissociation (CAD). QQQ scans were acquired as MRM experiments with optimized decluttering potential (DP) and collision energy (CE) for each individual MRM transition [40]. The m/z range was set between 50 and 1000.

Metabolites were identified by searching internal databases and public databases (Mass Bank, KN Ap Sac K, HMDB, Mo to DB, and METLIN) and comparing the m/z values, RT, and fragmentation patterns with the standards [41].

### Metabolomic data analysis

Those with a *P* value for the T test of < 0.05 and VIP ≥ 1 were considered differential metabolites between those groups. We constructed metabolic pathways based on the information in the KEGG database.

### Combined metabolomic and transcriptomic analysis

To reveal the regulatory and influencing mechanism between gene expression and metabolites, we analysed three models based on gene expression and metabolite abundance. The correlation between the top 250 differentially expressed genes and their metabolites was used to draw a heatmap.

## Supplementary Information

**Additional file 1: Figure S1.** Heatmap of the top 250 DEGs and their metabolites.

**Additional file 2: Table S1.** qPCR primer information.

**Additional file 3: Table S2.** Number of metabolites.

## Data Availability

The Sequence dataset used and/or analyzed during the current study are available from the corresponding author on reasonable request.

## References

[1] Williams B, Tudzynski B, Tudzynski P, Kan JAL (2007). *Botrytis cinerea*: the cause of grey mold disease. Mol Plant Pathol.

[2] Veneault FC, Barooah M, Egan M, Wakley G, Talbot NJ (2006). Autophagic fungal cell death is necessary for infection by the rice blast fungus. Science.

[3] Dean R, Van KJA, Pretorius ZA, Hammond KE, Di PA, Spanu PD (2012). The top 10 fungal pathogens in molecular plant pathology. Mol Plant Pathol.

[4] Wang WW, Zheng C, Hao WJ, Ma CL, Ma JQ, Ni DJ, Chen L (2018). Transcriptome and metabolome analysis reveal candidate genes and biochemicals involved in tea geometrid defense in *Camellia sinensis*. PLoS One.

[5] Wang XJ, Zhang X, Yang JT, Wang ZX (2018). Effect on transcriptome and metabolome of stacked transgenic maize containing insecticidal cry and glyphosate tolerance epsps genes. Plant J.

[6] Zhang Y, Li DH, Zhou R, Wang X, Dossa K, Wang LH, Zhang Y, Yu J, Gong H, Zhang X, You J (2019). Transcriptome and metabolome analyses of two contrasting sesame genotypes reveal the crucial biological pathways involved in rapid adaptive response to salt stress. BMC Plant Biol.

[7] Castillo L, Plaza V, Larrondo LF, Canessa P (2017). Recent advances in the study of the plant pathogenic fungus Botrytis cinerea and its interaction with the environment. Curr Protein Peptide Sci.

[8] Adachi H, Ishihama N, Nakano T, Yoshioka M, Yoshioka H (2016). Nicotiana benthamiana MAPK-WRKY pathway confers resistance to a necrotrophic pathogen *Botrytis cinerea*. Plant Signal Behav.

[9] Han XF, Li S, Zhang M, Yang LY, Liu YD, Xu J, Zhang S (2019). Regulation of GDSL lipase gene expression by the MPK3/MPK6 Cascade and its downstream WRKY transcription factors in Arabidopsis immunity. Mol Plant Microbe Interact.

[10] Lu B, Wang YH, Zhang G, Feng YN, Yan ZM, Wu JH, Chen X (2019). Genome-wide identification and expression analysis of the strawberry *FvbZIP* gene family and the role of key gene FabZIP46 in fruit resistance to gray Mold. Plants (Basel).

[11] Hu ZJ, Shao SJ, Zheng CF, Sun ZH, Shi JY, Yu JQ, Qi Z, Shi K (2018). Induction of systemic resistance in tomato against *Botrytis cinerea* by N-decanoyl-homoserine lactone via jasmonic acid signaling. Planta..

[12] Liu XT, Cao XQ, Shi SC, Zhao N, Li DD, Fang PH, Chen X, Qi W, Zhang Z (2018). Comparative RNA-Seq analysis reveals a critical role for brassinosteroids in rose (*Rosa hybrida*) petal defense against *Botrytis cinerea* infection. BMC Genet.

[13] Qing L (2016). Study on 2,4-epibrassinolide induced grape resistance to downy mildew and gray mold. Northwest agricultural and Forestry University.

[14] Yang Y, Zhang ZM, Li ML, Zhao LY, Jin P, Zheng YH (2019). The action modes of 2,4-epibrassionolide on controlling gray mold decay in postharvest grapes. Food Sci.

[15] Nie PP, Chen C, Yin Q, Jiang CH, Guo JH, Zhao HW, Niu D (2019). Function of miR825 and miR825* as negative regulators in Bacillus cereus AR156-elicited systemic resistance to *Botrytis cinerea* in *Arabidopsis thaliana*. Int J Mol Sci.

[16] Chen LM, Wu QC, He TJ, Lan JJ, Ding L, Liu TF (2010). Transcriptomic and Metabolomic Changes Triggered by *Fusarium solani* in Common Bean (*Phaseolus vulgaris* L.). Genes.

[17] Muñoz M, Faust JE, Schnabel G (2019). Characterization of *Botrytis cinerea* from commercial cut flower roses. Plant Dis.

[18] Han X, Kahmann R (2019). Manipulation of Phytohormone pathways by effectors of filamentous plant pathogens. Front Plant Sci.

[19] Nejat N, Mantri N (2017). Plant Immune System: Crosstalk Between Responses to Biotic and Abiotic Stresses the Missing Link in Understanding Plant Defence. Curr Issues Mol Biol.

[20] Dodds PN, Athjen JP (2010). Plant immunity: towards an integrated view of plant-pathogen interactions. Nat Rev Genetic.

[21] Couto D, Zipfel C (2016). Regulation of pattern recognition receptor signaling in plants. Nat Rev.

[22] Dangl JL, Horvath DM, Staskawicz BJ (2013). Pivoting the plant immune system from dissection to deployment. Science..

[23] Asai S, Mase K, Yoshioka H (2010). A key enzyme for flavin synthesis is required for nitric oxide and reactive oxygen species production in disease resistance. Plant J.

[24] Thomma BP, Eggermont K, Penninckx IA, Mauch MB, Vogelsang R, Cammue BP (1998). Separate jasmonate-dependent and salicylate-dependent defense-response pathways in Arabidopsis are essential for resistance to distinct microbial pathogens. Proc Natl Acad Sci U S A.

[25] Ren HR, Bai MJ, Sun J, Liu JJ, Liu JY, Ren M (2020). RcMYB84 and RcMYB123 mediate jasmonate-induced defense responses against Botrytis cinerea in rose (Rosa chinensis). Plant J.

[26] Tian Y (2013). Cloning and characterization of apple disease resistant-related genes *MdTGA2.1*, *MdAP2D4*, and *MdAP2D19*.

[27] Ng DW, Abeysinghe JK, Kamali M (2018). Regulating the regulators: the control of transcription factors in plant defense signaling. Int J Mol Sci.

[28] Zaynab M, Fatima M, Abbas S, Sharif Y, Umair M, Zafar MH, Bahadar K (2018). Role of secondary metabolites in plant defense against pathogens. Microb Pathog.

[29] Clay NK, Adio AM, Denoux C, Jander G, Ausubel FM (2009). Glucosinolate metabolites required for an Arabidopsis innate immune response. Science..

[30] Stotz HU, Sawada Y, Shimada Y, Hirai MY, Sasaki E, Krischke M, Brown PD, Saito K, Kamiya Y (2011). Role of camalexin, indole glucosinolates, and side chain modification of glucosinolate-derived isothiocyanates in defense of Arabidopsis against Sclerotinia sclerotiorum. Plant J.

[31] Cao XQ, Yan HJ, Liu XT, Li DD, Sui MJ, Wu J, Yu H, Zhang Z (2019). A detached petal disc assay and virus-induced gene silencing facilitate the study of Botrytis cinerea resistance in rose flowers. Horticulture Research.

[32] Zhong SL, Joung JG, Zheng Y, Chen YR, Liu B, Shao Y (2011). High-throughput illumina strand-specific RNA sequencing library preparation. Cold Spring Harbor Protocol.

[33] Kim D, Langmead B, Salzberg SL (2015). HISAT: a fast spliced aligner with low memory requirements. Nat Methods.

[34] Pertea M, Pertea GM, Antonescu CM, Chang TC, Mendell JT, Salzberg SL (2015). StringTie enables improved reconstruction of a transcriptome from RNA-seq reads. Nat Biotechnol.

[35] Pertea M, Kim D, Pertea GM, Leek JT, Salzberg SL (2016). Transcript-level expression analysis of RNA-seq experiments with HISAT, StringTie and Ballgown. Nat Protoc.

[36] Love MI, Huber W, Anders S (2014). Moderated estimation of fold change and dispersion for RNA-seq data with DESeq2. Genome Biol.

[37] Kanehisa M, Goto S (2000). KEGG: Kyoto encyclopedia of genes and genomes. Nucleic Acids Res.

[38] Zhang Z, Thomma BP (2014). Virus-induced gene silencing and agrobacterium tumefaciens-mediated transient expression in Nicotiana tabacum. Methods Mol Biol.

[39] Chen W, Gong L, Guo ZL, Wang WS, Zhang HY, Liu XQ, Yu S, Xiong L, Luo J (2013). A novel integrated method for large-scale detection, identification, and quantification of widely targeted metabolites: application in the study of rice metabolomics. Mol Plant.

[40] Wishart DS, Jewison T, Guo AC, Wilson M, Knox C, Liu Y, Djoumbou Y, Mandal R, Aziat F, Dong E, Bouatra S, Sinelnikov I, Arndt D, Xia J, Liu P, Yallou F, Bjorndahl T, Perez-Pineiro R, Eisner R, Allen F, Neveu V, Greiner R, Scalbert A (2013). HMDB 3.0—the human metabolome database in 2013. Nucleic Acids Res.

[41] Zhu ZJ, Schultz AW, Wang J, Johnson CH, Yannone SM, Patti GJ, Siuzdak G (2013). Liquid chromatography quadrupole time-of-flight mass spectrometry characterization of metabolites guided by the METLIN database. Nat Protoc.

