# Supplementary figures and images for: RcTGA1 and glucosinolate biosynthesis pathway involvement in the defence of rose against the necrotrophic fungus Botrytis cinerea
Source: BMC Plant Biol. 2021 May 17;21:223. doi: 10.1186/s12870-021-02973-z (PMC8130329; doi:10.1186/s12870-021-02973-z)

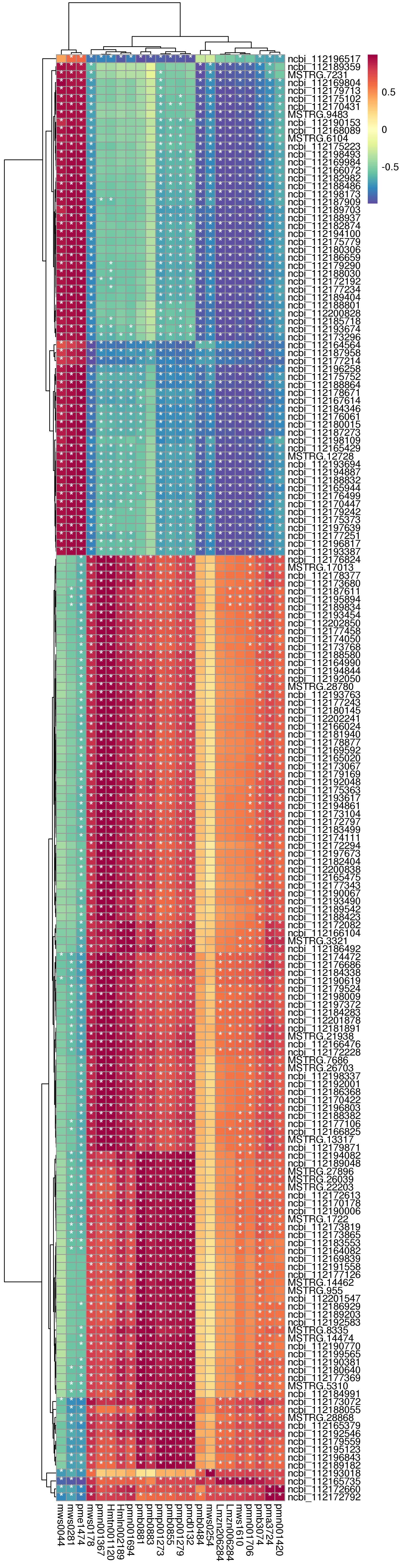

Supplement: Supplementary file 1 — Additional file 1: Figure S1. Heatmap of the top 250 DEGs and their metabolites. [file 12870_2021_2973_MOESM1_ESM.png]
